# Imaging application in ventricular arrhythmia of ischemic cardiomyopathy: diagnosis, treatment and prognosis

**DOI:** 10.1186/s44156-025-00082-3

**Published:** 2025-09-22

**Authors:** Fengli Hu, Ting Tang, Pengfei Wang, Guoqiang Gu, Ling Xue

**Affiliations:** Department of Cardiology, The Second Hospital of Hebei Medical University, Shijiazhuang, Hebei China

**Keywords:** Ventricular arrhythmia, Ischemic cardiomyopathy, Sudden cardiac death, Imaging

## Abstract

Ventricular arrhythmia (VA) is one of the common complications of many heart diseases in clinical practice, even some of its clinical symptoms are mild and non-specific, but the other may lead to sudden cardiac death (SCD) and most life-threatening VA is associated with ischemic cardiomyopathy (ICM). Nowadays, the developments in imaging techniques have provided clues to identify these highly variable VAs, which make clinicians identify patients with VA early and effectively who may have fatal consequences. Thereafter, it is beneficial to manage the risk stratification of patients, optimize their follow-up treatment, and improve clinical outcomes. This article reviews current ultrasound and magnetic resonance imaging techniques that can aid in diagnosis, treatment and prognosis, and provides clinicians with practical imaging and analytical recommendations to further identify patients with ICM who may develop VA.

**Clinical trial number** Not applicable.

## Background

Ventricular arrhythmias (VA) include premature ventricular contraction, ventricular tachycardia, ventricular flutter, and ventricular fibrillation, which may have no specific symptoms but may lead to sudden cardiac death (SCD) in severe cases, and most life-threatening VA is associated with ischemic cardiomyopathy (ICM), particularly in older patients [1]. In addition, autopsy of the SCD population revealed that ischemic heart disease accounted for 74.8% of the population, and the risk of SCD was increased by approximately 50% in patients with prior myocardial infarction (MI) compared with those without [2]. These results suggest that it is important to carry out timely and effective diagnosis, treatment and risk assessment of ICM-related VA.

The induction of ventricular arrhythmias often involves increased automaticity, triggered activity, and reentry, but the occurrence of VA in patients with ICM is often associated with triggered activity and reentry. It reported that the infarcted myocardium is prone to early post-depolarization which may lead to triggered activity, while the heterogeneity of normal myocardium, scarring, and border area may cause reentry [3]. In addition, the autonomic innervation disorder at the infarct site is also prone to VA [3]. Infarcted myocardium and infarct border zone (BZ) in ICM patients are associated with an increased risk of reentrant arrhythmias, thus accurate analysis of myocardial scars and border zones (quality and channel analysis) can help predict the risk of arrhythmias in patients with myocardial infarction [4].

At the same time, the advancement of cardiac imaging technology can not only provide information to assist in diagnosis, but also help to select treatment regimens, especially the development of ablation therapy often depends on imaging technology to provide proarrhythmic matrix information, so as to ensure the safety and effectiveness of ablation treatment. Therefore, the identification of normal and abnormal myocardium is helpful for the diagnosis, treatment and the evaluation of patient prognosis. In addition, VA risk stratification in ICM patients can assist in prognostic judgment and facilitate long-term management. In summary, this article aims to review the application of ultrasound and magnetic resonance imaging techniques in the diagnosis, treatment and prognostic evaluation of ischemic cardiomyopathy and ventricular arrhythmia, and to provide reference for clinical decision-making and choice. The relevant content is summarized in Table 1.

**Table 1:** Imaging technologies applied to evaluate ventricular arrhythmia in ischemic cardiomyopathy patients

Imaging technologies	Modalities	Parameters	Advantages	Disadvantages
Echocardiography	2D echocardiography	LVEF, WMSI, RWT	Convenient, No radiation exposure	Low resolution
	Strain echocardiography	GLS, GCS, GRS, GAS, MD, IBS		
CMR	LGE-CMR	IS, BZ mass, BZC, BZC mass, Grey zone	High resolution	Time-consuming
	Mapping	T1 mapping, T2 mapping		
	Strain evaluation	GLS, GCS, GRS, GAS, MD		

CMR, cardiac magnetic resonance; LGE-CMR, late gadolinium enhancement-cardiac magnetic resonance; LVEF, left ventricular ejection fraction; WMSI, wall motion score index; RWT, relative wall thickness; GLS, global longitudinal strain; GCS, global circumferential strain; GRS, global radial strain; GAS, global area strain; MD, mechanical discrete; IBS, integrated backscatter; IS, infarct size; BZ mass, border zone mass; BZC, border zone channel; BZC mass; border zone channel mass

## Ultrasound

### Echocardiography

Echocardiography is a non-invasive examination method for evaluating the structure and function of the heart [5]. Currently, the most widely used is transthoracic echocardiography, which is used for valvular disease, coronary heart disease, dilated cardiomyopathy, and hypertrophic cardiomyopathy [5]. It is a first-line tool for diagnosis and risk stratification of diseases such as cardiomyopathy and arrhythmogenic right ventricular cardiomyopathy [5]. Echocardiography has not only become the best non-invasive examination method currently used to evaluate patients with different types of cardiovascular diseases, but it is also the first imaging tool widely used to guide management strategies for ventricular arrhythmias [6]. It provides important information for the diagnosis, treatment selection and prognosis assessment of ischemic cardiomyopathy-related ventricular arrhythmias.

The latest european society of cardiology (ESC) guidelines recommend ICM patients with left ventricular ejection fraction (LVEF) ≤ 35% and New York Heart Association (NYHA) class II and III implant implantable cardioverter defibrillator (ICD) for primary prevention [6]. Although reduced LVEF is associated with VA and mortality in ICM patients, its risk stratification ability is not so good. Some patients after myocardial infarction with LVEF > 35% still develop SCD [7], which has led to many studies devoted to finding better evaluation indicators.

In addition to LVEF, many other echocardiographic indicators can provide prognostic information in patients with cardiac disease and assist in risk stratification. Among them, the wall motion score index (WMSI) can evaluate the degree of abnormal wall motion in patients with ischemic cardiomyopathy, distinguish ischemic and non-ischemic causes, and predict adverse outcomes [7]. Currently, a 16-segment model is commonly used to score ventricular wall motion (normal motion is 1, reduced motion is 2, motion disappears is 3, and paradoxical motion is 4), WMSI = Sum of motion score of each ventricular wall segment/total segments [8]. The study found that in coronary heart disease patients who implanted with an ICD for secondary prevention, an overall WMSI ≥ 1.5 can identify ICD events (including ICD-related antiarrhythmic pacing or timely ventricular arrhythmia shocks recorded by the ICD) [9]. It also differentiated the patients who died from all causes (*P =* 0.008), and for every 1-point increase in overall WMSI, the risk of events doubled (hazard ratio: 2.18, 95%CI: 1.03–4.65, *P =* 0.04) [9].

Another indicator is relative wall thickness (RWT), which is defined as twice the posterior wall thickness divided by the left ventricular diastolic diameter [7]. It can reflect the geometry of the left ventricle and is mainly used to evaluate left ventricular weight. structure, and can distinguish centripetal and centrifugal remodeling [7]. High RWT reflects concentric remodeling and is associated with an increased risk of mortality in patients with hypertrophic cardiomyopathy [10]. A study including 1260 patients in the MADIT-cardiac resynchronization therapy (CRT) trial (including 88 ICM patients with LVEF ≤ 30% and indications for CRT) compared the echocardiographic parameters related to VA, which including RWT, left ventricular end-diastolic volume (LVEDV), left ventricular end-systolic volume, LVEF and LV mass. Ultimately it was found that RWT is the best parameter to predict VA [10]. Patients with low RWT (*<* 0.24) have an 83% increased VA risk (*P <* 0.001), while every 0.01 unit decrease in RWT the VA risk increases by 12% ( *p* < 0.001) [10]. Although this study found a correlation between RWT and VA risk, ICM patients were not analyzed separately. Considering that RWT mainly reflects ventricular remodeling, it may be more valuable for ICM patients with left bundle branch block who require CRT treatment.

Echocardiography is convenient, fast and has no radiation exposure. Currently, LVEF is widely used in clinical practice and has been identified as a VA/SCD risk assessment indicator for ICM patients. However, the ability of LVEF to identify ICM patients with high VA risk is limited, and we hope to find indicators with higher specificity. Some studies have confirmed that indicators such as WMSI and RWT can predict VA risk in ICM patients, while it is important to note that these indicators may not be uniformed, and large randomized prospective studies are needed to confirm its efficacy. In addition, echocardiography-related indicators are based on the assessment of global left ventricular function and may miss local lesion characteristics, which is particularly important for ICM-related VA. As a result, strain echocardiography has emerged, which can assess local strain and predict VA risk in patients with ICM.

### Strain echocardiography

**Fig. 1:**
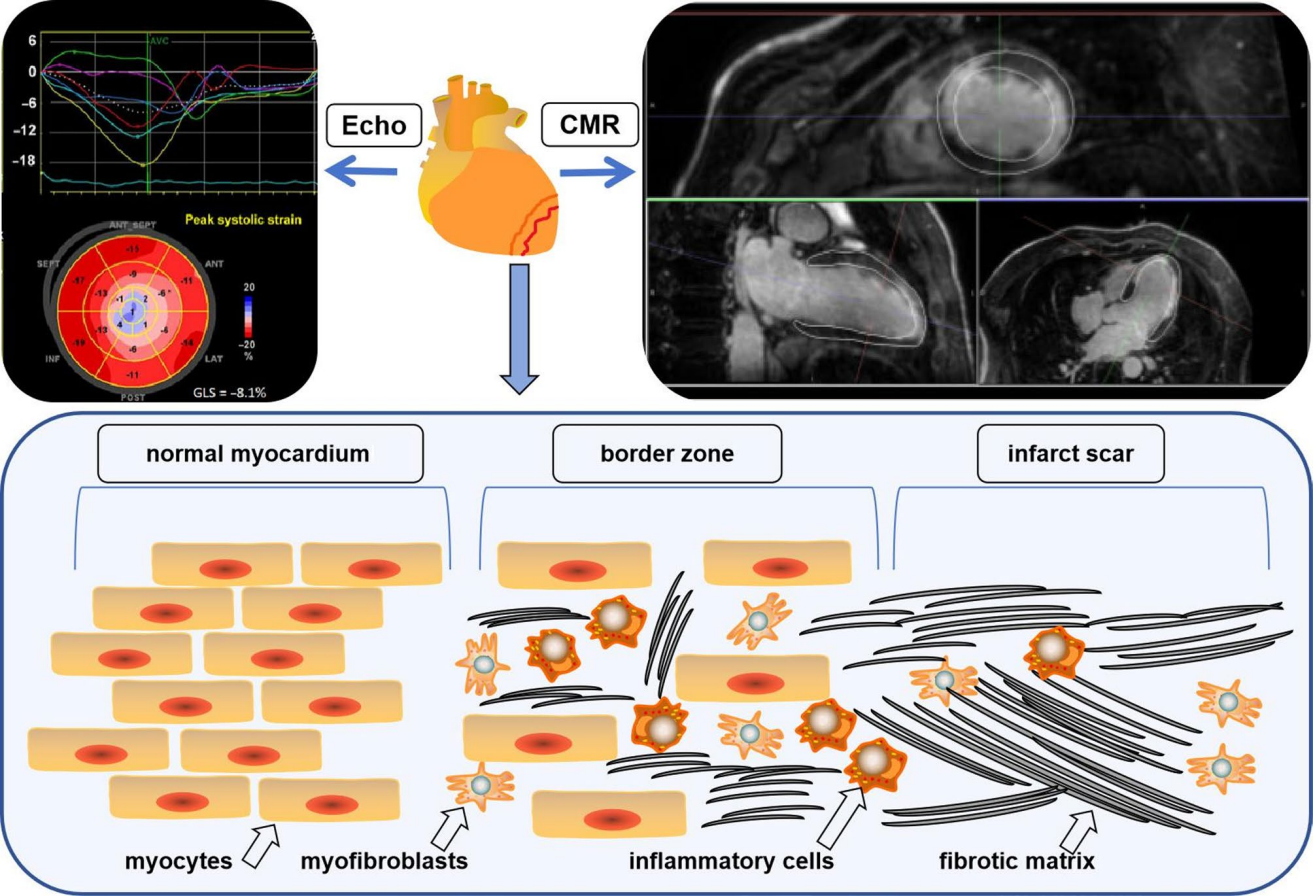
Imaging technologies applied to evaluate ventricular arrhythmia in ischemic cardiomyopathy patients. Echo, Echocardiography; CMR, cardiac magnetic resonance

Strain echocardiography can quantitatively assess left ventricular and global myocardial function in multiple planes and directions [11]. It can specifically measure global/segmental longitudinal strain (GLS/SLS), global/segmental radial strain (GRS/SRS), global/segmental circumferential strain (GCS/SCS), global/segmental area strain (GAS/SAS), mechanical discrete (MD)etc. In a word, it can assess global and local myocardial strain capacity especially postinfarct lesions in ICM patients, which is conducive to diagnosis and treatment and risk stratification [11]. A typical strain echocardiography of ICM patients is shown in Fig. 1 [11].

#### Global strain assessment

GLS and MD have been extensively studied in global strain assessment. GLS is the average peak systolic strain measured by speckle tracking technology, which can reliably assess left ventricular function and detect subtle changes in left ventricular function in patients with preserved LVEF [12]. And MD is defined as the standard deviation of the time from the onset of the Q/R wave of the electrocardiogram to the peak negative strain of each left ventricular segment, which can assess myocardial contractile heterogeneity [13].

Studies have found that global strain indicators can indicate the extent of myocardial infarction [14]. A meta-analysis including 765 MI patients confirmed that GLS has a good correlation with the extent of myocardial infarction displayed by cardiac magnetic resonance (CMR)(pooled *r* = 0.70, 95%CI: 0.64,0.74) [14]. GLS discriminated the > 12% MI areas with the sensitivity and specificity of 0.77, 0.86 respectively, and the area under curve (AUC) measured by the total receiver operating characteristic curve (ROC) was 0.702 [14]. Another study showed that in patients with acute myocardial infarction (AMI), there was a significant correlation between infarct size and average GLS, GLS, and LVEF no matter at admission or after revascularization (*P <* 0.0001) [15]. At the same time, average GLS had the best correlation and the best ability to distinguish different infarct size [15]. Using − 7.6% as cutoff value at admission, the sensitivity and specificity are: 89% and 88% respectively, and the AUC for identifying large-area infarction is 0.92 [15]. In addition, a study used late gadolinium enhancement-cardiac magnetic resonance (LGE-CMR) as the standard to evaluate the ability of MD in post-percutaneous coronary intervention (PCI) ST-segment elevation myocardial infarction (STEMI) patients (96 cases) and found that MD was significantly correlated with the infarct core area, total scar burden, and border zone (*r* = 0.517, 0.497, 0.298, all *P <* 0.001) [16].

Global strain indicators not only suggest myocardial scarring, but also assess prognosis. In patients with successful reperfusion after STEMI, GLS was an independent predictor of clinical outcome (a composite of death, hospitalization for heart failure, nonfatal MI, and VA) after 39 ± 19 months of follow-up (hazard ratio(HR) = 1.37, *P* = 0.001), with the best predictive ability for patients with LVEF of 40–50% (HR = 2.25, *P <* 0.001) [17]. A meta-analysis including 2634 ICM patients found that after 17–70 months of follow-up, patients with elevated MD had a higher risk of VA, and every 10 ms increase in MD was significantly and independently associated with VA (HR: 1.19; 95% confidence interval (CI): 1.09 to 1.29; *P <* 0.01) [13]. And another study found that patients with MD > 70ms (including ICM and Non-ICM patients with LVEF < 40%) had a higher VA risk than patients with MD < 70ms (Log rank *P <* 0.001), especially those with ICM who had MD > 70ms [18]. And the highest risk expressed an annual fatal VA incidence rate of 16% (Log rank *P <* 0.001) [18].

Otherwise compared with LVEF, global strain assessment often provides more prognostic information. A prospective, multicenter study including 569 patients with AMI (268 patients with STEMI and 301 patients with Non-STEMI) followed up and found that patients with sustained ventricular tachycardia/SCD had lower LVEF (48 ± 17% vs. 55 ± 11%, *P <* 0.01), GLS was lower (-14.8 ± 4.7% vs. -18.2 ± 3.7%, *P* = 0.001), and MD was higher (63 ± 25 ms vs. 42 ± 17 ms, *P <* 0.001) [19]. When evaluating patients with NSTEMI and LVEF > 35%, LVEF was not predictive of arrhythmia risk (*p* = 0.33), whereas MD and GLS were predictive (*P* < 0.05 for both) [19]. Combining MD and GLS to predict arrhythmia events can achieve the best positive predictive value of 21% (95%CI: 6–46%) [19]. The IMPROVE sub-study included 290 patients with LVEF < 40% (including 71% of ICM patients), and after follow-up, univariate analysis found that MD (*P <* 0.01), GLS (*P <* 0.01) and LVEF (*P =* 0.01) were fatal VA Predictor factors, while in multivariate analysis MD (HR: 1.02, *P =* 0.01) and GLS (HR: 1.14, *P =* 0.004) were still independent predictors of fatal VA, but LVEF failed to predict fatal VA (*P =* 0.14) [18].

Some studies have compared the predictive ability of GLS and MD on prognostic outcomes. Follow-up of 96 STEMI patients with PCI found that GLS and MD were sensitive to predicting composite endpoints (all-cause death, ICD response to VA/VA shock and anti-tachycardia pacing) [16]. And the sensitivities are 0.615, 0.923 respectively, even the specificities are: 0.895, 0.618 respectively. Further analysis found that the predictive ability of MD was better than that of GLS, although the difference between the two was not statistically significant (AUC = 0.847 vs. 0.822, *P >* 0.05) [16]. A multicenter, observational study on patients with LVEF ≤ 45% (of which 56% were ischemic patients) found that the predictive sensitivity and specificity of MD ≥ 75ms were: 91% and 79% [20]. When GLS ≥ -14% is used as the cut-off value, the sensitivity and specificity are: 90% and 19% [20]. After Cox multivariate analysis, it was confirmed that only MD ≥ 75ms is a predictor of VA events (HR: 9.45; 95%CI: 4.75 to 18.81; *P <* 0.0001) [20]. Another study found that MD (HR: 1.02, *P =* 0.01) and GLS (HR: 1.14, *P =* 0.004) were independent predictors of fatal VA in patients with ICM after 22 ± 12 months of follow-up, and after adjusting for age, gender, atrial fibrillation and left ventricular systolic volume (LVESV), MD remained independently predictive of fatal VA (HR: 1.19; 95%CI: 1.08–1.32, *P* = 0.001) [18].

#### Local strain assessment

The global strain assessment may omit local lesion information, and some studies suggest that local strain damage is a more accurate diagnostic and prognostic indicator than the global strain assessment. A study of 1064 patients enrolled in the MADIT-CRT trial (including 592 patients with ICM) found that patients with VA had lower LVEF compared with those without VA after a mean follow-up of 2.9 years (28.3% vs. 29.5%; *P <* 0.001) [21]. And the patients with VA had lower longitudinal strain (anterior-strain, -7.7% versus − 8.8%; *P <* 0.001; lateral-strain, -7.3% versus − 7.9%; *P =* 0.022; inferior-strain, -8.3% versus − 9.9%; *P <* 0.001; septal-strain, -9.1% versus − 10.0%; *P <* 0.001) [21]. Moreover, the longitudinal strain of the anterior and inferior walls remained independent predictors of VA after multivariate adjustments (anterior: HR, 1.08 (1.03–1.13); *P =* 0.001; inferior: HR, 1.08 (1.04–1.12); *P <* 0.001; per 1% absolute decrease for both) [21]. Besides the risk of VA was increased by 2 folds in patients with inferior strain *<* 7% (HR, 2.1 (1.63–2.69), P *<* 0.001) [21]. However, the ability of MD to predict VA risk was not found in the study, and given the study did not use a commonly used 16-segment model to measure MD it may lead to inconsistent results.

In another study of 467 patients with prior MI and LVEF *>* 35%, the strain of patients with VA events was significantly impaired compared with those without VA events, with the sensitivity of GLS (-12.7% cut-off) and MD (61 ms cut-off) predicting VA of 77%, 85%, and specificity of 66%, 73% respectively [22]. Multivariate analysis showed that MD (*P =* 0.01), posterior inferior circumferential strain (*P =* 0.04), and marginal circumferential strain (*P =* 0.002) were independently associated with arrhythmic events [22]. Besides, the strain value of posterior inferior infarction was more correlated with arrhythmic events than global strain, and circumferential strain around infarction was the strongest predictor of ventricular arrhythmia [22].

These suggest that ICM patients should pay attention to the changes in local strain (especially the area associated with myocardial infarction) while assessing the overall strain, which may provide more prognostic information and provide possible targets for the diagnosis and intervention of VA in ICM patients.

#### 3D-STE

Three-dimensional speckle tracking echocardiography (3D-STE) can obtain three-dimensional full-volume imaging to track myocardial acoustic spot movement in three-dimensional space and evaluate cardiac function more intuitively and accurately [23].

3D-STE is useful in assessing myocardial fibrosis. In a study of 50 patients who examined LGE-CMR and 3D-STE at the same time, when the cut-off value of GLS *>* -15.25%, 3D GLS was associated with myocardial fibrosis as defined by LGE (10% LGE is myocardial fibrosis), with sensitivity and specificity of 84.6% and 84.8%. But the sample in this study was small, in which only 4 of the 29 patients defined scar by LGE-CMR [24]. In 100 successfully revascularized STEMI patients, CMR and echocardiography were used to evaluate cardiac function, and the 3D strain indexes (including GLS, GCS, GRS, GAS) were correlated with the range of transmural infarction (LGE *>* 50%) (with all correlation coefficients *P <* 0.0001) [25]. Further segmental analysis showed that the segmental 3D strain index had a good predictive ability for the non-viable segment (LGE *>* 75%) (the AUC of segments LS, CS, RS, and AS were 0.752, 0.807, 0.823, and 0.824 respectively) [25].

It is reported that 3D-STE is better than 2D-STE. In a study of 110 patients with STEMI who underwent primary PCI therapy, 2D and 3D strain ultrasound evaluation within 24 h after surgery was performed with a 20% increase in left ventricular end-diastolic volume as the criterion for left ventricular remodeling [26]. And after 3 months of follow-up, 2D GLS (-12.5 ± 3.2% vs. -15.0 ± 3.1%, *P <* 0.001), 3D GLS (-9.9 ± 2.2% vs. -13.1 ± 2.7%, *P <* 0.001), 3D GAS (-20.3 ± 3.9% vs. -23.3 ± 4.8%, *P* = 0.005), and 3D GRS (29.0 ± 7.4% vs. 34.3 ± 8.5%, *P* = 0.007) all reduced significantly, while 3D GCS was not significantly different (-12.7 ± 2.9% vs. -13.0 ± 3.2%, *P* = 0.822) [26]. Further comparison of predictive power showed that 3D GLS was the most powerful predictor, and 3D GLS was better than 2D GLS (AUC 0.82, 0.72, *P* = 0.034), and better than 3D GAS and 3D GRS (both AUC = 0.68, *P <* 0.001) [26]. In the same study focusing on STEMI patients, echocardiography was performed within 48 h of PCI, and after an average follow-up of 118 months, it was found that 3D indicators were better than 2D indicators in predicting cardiac death and heart failure: 3D-GLS *>* 2D-GLS (AUC 0.869, 0.808), 3D-MD *>* 2D-MD (AUC 0.710, 0.655, respectively), while multivariate analysis showed that 2D-GLS *>* -11.2% (HR: 5.947, CI: 3.037–11.631, p *<* 0.0001), 3D-GLS*>*-11.3% (HR: 10.656, CI: 4.031–17.131, *P <* 0.0001), 3D-MD *>* 56.7ms (HR: 1.991, CI: 1.033–3.613, *P =* 0.03) were independent predictors of cardiac death and heart failure [27]. The combination of 3D-GLS and 3D-MD had the best predictive power for cardiac death and heart failure (log rank χ2 = 94.1, *P <* 0.0001) [27].

However, a recent study found that 3D strain prediction was slightly lower than that of 2D strain [28]. It included 545 STEMI patients who received revascularization (including PCI and thrombolytic therapy for contraindications to exclusion) and after a mean follow-up of 49.5 months it was found that 2D EF, 2D GLS, 3D EF, 3D GLS and 3D GAS are significantly associated with adverse outcomes (all-cause death or readmission to VA/acute heart failure) (all p *<* 0.001) [28]. The predictive ability of GLS on adverse outcomes in 3D global strain parameters was better than that of GAS, GRS, GCS, etc. (AUC = 0.6684, 0.6525, 0.6481, 0.6197 respectively), but the difference was not statistically significant [28]. At the same time, there was no significant difference between 2D EF (AUC = 0.6988) and 2D GLS (AUC = 0.7075) (*P =* 0.394, 0.195 respectively). However, comparing predictive power, the likelihood ratio for 2D GLS was 15.9 (*P <* 0.001), while 3D GLS was 1.49 (*P* = 0.22) [28]. However, it is important to note that although the study defined follow-up outcomes as including all-cause death and readmission due to acute decompensated heart failure/VA, all-cause mortality was 7.5% for the follow-up outcomes and 2.6% for readmission due to acute decompensated heart failure, and no readmission due to VA was identified. Besides, the study included only STEMI patients with sinus rhythm, which reduced the possibility of finding a correlation between 3D strain measures and VA [28].

#### Assessment of tissue characteristics

In addition, the ultrasound integrated backscatter (IBS), a parameter that measures myocardial ultrasound reflex in decibels based on dot tracking technology to assess myocardial tissue characteristics, has been shown to be useful for the assessment of myocardial fibrosis [29]. Tissue characteristics assessed using IBS have been found to predict myocardial fibrosis on endocardial biopsy in patients with dilated cardiomyopathy [30], while posterior IBS was found to be higher in patients with ICM (13.2 versus − 4.4 versus 9.2 versus − 2.4 dB; *P =* 0.002) [31]. Besides a study of ICM patients with ICD found that patients with VA and arrhythmic storms had higher apical-septal and apical-lateral segment-corrected IBS (0.66 ± 0.11 vs. 0.50 ± 0.16, *P* = 0.008; 0.62 ± 0.19 vs. 0.46 ± 0.18, *P* = 0.041) [32]. Although echocardiography-based techniques have the advantages of convenience and speed, IBS is rarely used to characterize myocardial tissue in clinical practice, and more studies are needed to confirm the ability of IBS to assess the risk of VA in ICM patients.

## CMR

CMR is the gold standard for quantifying the structure and function of the heart chambers due to its good soft tissue image resolution, which can detect myocardial edema, fibrosis, infiltration, and perfusion defects, and can be quantitatively evaluated in combination with the structure and function of the heart chambers [33]. Cardiac structure and function can be evaluated, and different sequences and processing software can evaluate blood flow and coronary circulation, while myocardial activity can be assessed and tissue quantitative assessment can be carried out for ICM patients, which is helpful for VA diagnosis and treatment and risk assessment.

### LGE-CMR (assessment of myocardial viability)

Myocardial infarction leading to myocardial membrane rupture and chronic interstitial dilation can both increase the volume of gadolinium‑ ethoxybenzyl‑ diethylenetriamine pentaacetic acid (Gd-DTPA) distribution in the myocardium, which allows late gadolinium enhancement (LGE) to assess myocardial viability and clearly visualize myocardial scarring and fibrosis [34]. The identification of myocardial lesions by LGE is helpful in ablation therapy, and expert’s consensus recommends that ischemia and non-ischemic patients undergo LGE-CMR prior to VA ablation [13]. In a study of 84 patients who were treated for ablation of scar-related monomorphic VA, CMR-guided VA ablation was found to be safe and feasible, significantly shortening the time of surgery, fluoroscopy, and radiofrequency, and found that CMR-guided ablation improved ablation triggered VA (18 verse. 46%, *P =* 0.04), and fewer VA recurrences after one year follow-up (log rank: 0.019), while no significant difference was found in intraoperative complications [35]. In addition, LGE-CMR can accurately define epicardial and intraventricular scars, which can help identify patients who benefit from epicardial ablation [36]. In the meanwhile, it can significantly improve prognosis, and accurate assessment of intraventricular scars can suggest the use of specific ablation techniques to improve ablation outcomes [36].

#### Scar assessment

Myocardial scarring found in LGE was associated with the occurrence of VA. After a follow-up of 3.36 ± 2.22 years in 147 patients with coronary atherosclerotic heart disease, it was found that the infarct size (IS) measured by CMR ≥ 35%, LVEF ≤ 31%, and WMSI ≥ 1.9 were strongly associated with the occurrence of cardiac events (myocardial infarction, heart failure readmission, fatal arrhythmias, and cardiac death) (P *<* 0.001) [37]. In a meta-analysis comparing positive MRI-LGE and adverse cardiovascular events in patients with ICM and Non-ICM, patients with positive LGE were associated with an increased risk of life-threatening arrhythmias (odds ratio (OR): 4.6; 95%CI: 2.7-8.0) and an increased risk of major adverse cardiovascular events (MACE)(OR: 4.7; 95%CI: 2.8–7.9) [38].

Moreover, assessment of myocardial scarring can help predict the risk of VA in patients with ICM. In a meta-analysis of eight prospective studies, LGE was found to predict VA risk in ICM patients with ICD, with a pooled sensitivity of 0.79 (95%CI: 0.66–0.87) and a specificity of 0.28 (95%CI: 0.14–0.46) [39]. Another study analyzed 30 patients with myocardial infarction with complete electrophysiological and cardiac MRI data, and found that patients with malignant arrhythmias had lower LVEF and higher scar quality (all *P <* 0.05), and regression analysis showed that LVEF (OR = 1.580) and scar quality (OR = 6.270) were risk factors for malignant VA after myocardial infarction. In addition, the predictive ability of scar quality for malignant VA was better than that of LVEF (AUC 0.839 and 0.696, respectively), while the combined scar mass and LVEF increased AUC to 0.848, and the sensitivity and specificity for predicting malignant VA were 0.688 and 0.857 [40].

#### Border zone

Color-coded pixel signal intensity (PSI) was used to distinguish myocardium into the core zone, the border zone (BZ) and healthy tissues (with 40% ± 5% and 60% ±5% of the maximum PSI as thresholds) [41]. Additionally, the border zone channel (BZC) is defined as a contiguous channel that connects two normal tissues and includes a scar core or anatomical barrier (i.e. mitral annulus) [41]. Nowadays more and more studies have found that the infarct border zone features found in LGE can better assess the risk of VA in ICM patients, and can provide better prognostic value than LVEF and overall scar characteristics.

An increase in BZ was found to be associated with an increased risk of VA. A prospective study of 74 ICM patients (LVEF ≤ 35%) who underwent ICD implantation, after a 5.4 ± 1.9 years follow-up, found that lower LVEF (HR: 0.92; *P* = 0.03), high left ventricular end-diastolic volume index (LVEDVi) (HR 1.02; *P <* 0.01), and larger border zone (HR 1.11, *P <* 0.01) is associated with VA [42]. Moreover, the border zone provides more prognostic information than LVEF. A study included 126 patients with STEMI observed that there was no significant difference in LVEF between the VA group and the control group (51.0 ± 11.6% vs. 55.5 ± 8.5%, *P =* 0.10), but the mean BZ quality was higher in VA patients (17.2 ± 10.3 g vs. 10.3 ± 6.0 g; *P* = 0.0002) with more BZ channels (17 (80%) vs. 44 (42%); *P* = 0.001) [43]. And further study confirmed the patients with a BZ mass ≥ 17.2 g and BZC had the highest risk of VA (OR: 9.4, 95%CI: 3.26–27.13, *P* ≤ 0.0001) [43]. In addition, in a retrospective study of 979 patients with coronary heart disease, after a mean follow-up of 5.82 years, it was found that the predictive power of gray zone fibrosis (GZF) ≥ 5.0 g on LGE was significantly better than that of LVEF (AUC 0.85, 0.75, *P =* 0.001), and GZF was closely associated with arrhythmia endpoint (sHR: 9.36; 95%CI: 5.42–16.1) [44].

Furthermore, border zone features were better predictors of VA risk than overall scar characteristics. A retrospective analysis of 82 ICM patients treated with ICD found a significant increase in total scar mass (60.0 g vs. 43.3 g, *P* = 0.009) in patients treated with timely ICD (ICD shock and anti-tachycardia pacing) [45]. Simultaneously, both total scar mass (HR: 1.02, 95%CI: 1.00-1.04, *P* = 0.014) and BZ mass (HR: 1.04, 95%CI: 1.01–1.07, *P* = 0.009) independently predicted timely ICD therapy [45]. In another study of 217 patients with an indication for CRT (39.6% ischemia), BZ mass (HR 1.06, 95%CI 1.04–1.08, *P <* 0.001) and BZC mass (HR 1.21, 95%CI 1.10–1.32, *P <* 0.001) were the strongest predictors of ICD therapy/SCD [46].

A recent study included 150 patients with MI (66 were VA seizure patients, matched 84 as the control group) and found that VA patients could be distinguished by a BZC mass of 5.15 g, with sensitivity and specificity of 92.4% and 86.9% respectively [41]. After adjusting for age, sex, and LVEF, BZC quality was the most relevant variable for the development of persistent monomorphic VA after MI (AUC: 0.93, CI: 0.89–0.97, *P <* 0.001), and significantly increased AUC compared with other scar parameters which covered total scar mass, BZ mass and core mass ( *P <* 0.001 for all pairwise comparisons) [41].

In conclusion, LGE can provide more comprehensive and accurate lesion information, which can help optimize patient risk stratification, and has the potential to become a new ICD indication evaluation indicator. However, it is noted that even LGE-CMR has been shown to be safe in patients with ICDs [47], artifacts may affect the quality of LGE-CMR images, and based on recent reports that the use of a new broadband sequence, LGE-CMR, can reduce or eliminate these artifacts to better interpret image results [48].

### Mapping technology (quantitative assessments)

Diffuse fibrosis/infiltrate, fatty deposition, iron deposition, and edema can be further quantitatively assessed using natural T1, T2, and T2 ∗, and accurate tissue localization and quantitative assessment can facilitate comprehensive analysis of lesions and may be a more favorable prognostic indicator than LGE [49].

Among them, T1 mapping can distinguish between irreversible and reversible myocardial injury in patients with STEMI by quantifying diffuse fibrosis, and T1 values are strongly correlated with left ventricular remodeling [50]. A prospective longitudinal study found that quantitative myocardial tissue assessment by T1 mapping in 130 patients (71 ICM) with ICD implantation can predict the risk of outcome events (included timely ICD treatment or documented ongoing VA) in ischemic and non-ischemic cardiomyopathy (HR 1.10; CI 1.04–1.16; 90-ms difference between the end point-positive and end point-negative groups) [51]. In patients with gadolinium contraindications, native T1 can be used to some extent to assess infarction, but due to its low specificity, it may not be reliable to infer infarction using this method alone [52].

Because both acute and chronic myocardial infarctions increase extracellular space, LGE imaging cannot distinguish between acute (necrosis) and chronic (scarring) myocardial infarction, and T2-mapping, which provides a better noninvasive assessment of myocardial edema than traditional T2-weighted imaging, can distinguish between necrosis and scarring [34].

Although mapping imaging can provide quantitative tissue assessment, LGE is currently mainly used for myocardial scar assessment, and more studies are needed to confirm its efficacy in assessing patients with VA risk ICM.

### Strain CMR

At present, the preferred technique for myocardial strain assessment with CMR is CMR magnetic resonance-feature tracking (CMR-FT) [53], which has been confirmed to be helpful in the prognostic risk estimation of ICM patients. In a study of 1235 patients, myocardial strain assessment based on CMR-FT demonstrated that myocardial strain assessment based on CMR-FT could stratify the risk of patients after MI, and the overall strain parameters including GCS, GRS, and GLS were significantly correlated with LVEF and infarction degree, among which GLS *>* 13.2% was the best predictor of MACE (AUC: 0.70, 95%CI: 0.67 to 0.72, *P <* 0.001) [54].

In contrast, after a mean follow-up of 90 months in patients with STEMI, MD assessed by CMR-FT was found to indicate the extent of myocardial injury and independently predicted mortality and risk of SCD (HR, 1.39; 95%CI, 1.20–1.62; *P <* 001) [55]. However, due to the lack of prospective large-sample multicenter studies, the use of CMR for myocardial strain assessment has not been widely used.

## Conclusions

VA in ICM patients is a common and major problem that cannot be ignored in clinical practice, and its unique pathophysiological features, including the identification of proarrhythmic matrix, are of great significance for further treatment and prognostic evaluation. Therefore, it is utterly important to use imaging methods to help accurate and individualized assessment of patients with VA and ICM. While further stratifying the risk of patients, it assists in screening suitable treatment options, and provides a strong guarantee for the safety and effectiveness of ablation therapy. Ultrasound and magnetic resonance imaging are commonly used in clinical examination, combined with many indicators of current research to optimize the management of patient risk stratification, follow-up treatment and improve clinical outcomes, clinicians should conduct a comprehensive and systematic VA risk assessment of ICM patients, especially in combination with these common examination results.

## Data Availability

No datasets were generated or analysed during the current study.
